# Analysis of skeletal stem cells by renal capsule transplantation and *ex vivo* culture systems

**DOI:** 10.3389/fphys.2023.1143344

**Published:** 2023-03-29

**Authors:** Wei Hsu, Takamitsu Maruyama

**Affiliations:** ^1^ Forsyth Institue, Cambridge, MA, United States; ^2^ Faculty of Medicine of Harvard University, Harvard School of Dental Medicine, Boston, MA, United States; ^3^ Harvard Stem Cell Institute, Cambridge, MA, United States

**Keywords:** bone, cartilage, skeletogenic mesenchyme, cranial suture, tissue regeneration, calvaria, mesenchymal stem cell, cell-based therapy

## Abstract

Skeletal stem cells residing in the suture mesenchyme are responsible for proper development, homeostasis, and injury repair of the craniofacial skeleton. These naïve cells are programmed to differentiate into osteoblast cell types and mediate bone formation *via* an intramembranous ossification mechanism. The simplicity of this system also offers great advantages to studying osteoblastogenesis compared to the appendicular and axial skeletons. Recent studies utilizing genetically based cell tracing have led to the identification of skeletal stem cell populations in craniofacial and body skeletons. Although the genetic analysis indicates these cells behave like stem cells *in vivo*, not all of them have been thoroughly examined by stem cell isolation and stem cell-mediated tissue generation. As regeneration is an integral part of stem cell characteristics, it is necessary to further analyze their ability to generate tissue at the ectopic site. The establishment of an *ex vivo* culture system to maintain the stemness properties for extended periods without losing the regenerative ability is also pertinent to advance our knowledge base of skeletal stem cells and their clinical applications in regenerative medicine. The purpose of this review is to discuss our recent advancements in analyses of skeletal stem cells using renal capsule transplantation and sphere culture systems.

## Introduction

The craniofacial skeleton consists of neurocranium and viscerocranium which are formed from skeletogenic mesenchyme derived from both mesoderm and neural crest (Morriss-Kay and Wilkie, 2005). The viscerocranium is divided into calvarium and chondrocranium (Wilkie and Morriss-Kay, 2001). The calvarium, also known as the skull vault, consists of bones that are formed *via* intramembranous ossification (Hall, 1990). This process differs from the endochondral ossification in appendicular and axial skeletons for which prior formation of cartilage templates is required (Ornitz and Marie, 2002; Galea et al., 2021). The simplicity thus offers calvaria an ideal system to study osteoblast cells and osteoblast differentiation due to the lack of involvement of additional cell types, e.g., chondrocytes, in the ossification processes. Although the calvarial mesenchyme is developmentally programmed to become osteoblast cell types, genetic mutations, and signaling stimuli have been shown to induce ectopic chondrogenesis (Day et al., 2005; Hill et al., 2005; Maruyama et al., 2010; Maruyama et al., 2022a). The evidence of cell fate switch indicates the existence of genuine skeletal stem cells (SSCs), leading to the isolation and purification of mouse and human suture stem cells (SuSCs) from the calvarial mesenchyme (Maruyama et al., 2016; Maruyama et al., 2022a). As a result, the calvarium provides an outstanding system to study SSCs and their development and programming into skeletal lineages.

Cell tracing analyses using the genetic labeling approach have identified cell populations exhibiting unlimited self-renewal ability and capable of differentiating into at least one specialized cell type (Sacchetti et al., 2007; Zhou et al., 2014; Chan et al., 2015; Worthley et al., 2015; Zhao et al., 2015; Maruyama et al., 2016; Debnath et al., 2018). These naïve cells meet the modern stem cell definition compared to mesenchymal stromal cells (MSCs) capable of giving rise to mesenchymal-derived cell types *in vitro* (Friedenstein et al., 1970; Caplan, 1991; Bianco et al., 2008; Uccelli et al., 2008; Caplan and Correa, 2011). However, the recently identified cell populations have not always been assessed by tissue-forming/skeletogenic ability.

## Assessing regenerative characteristics of stem cells by *in vivo* tissue-forming ability

SSCs are rigorously defined as multipotent stem cells able to even generate bones and cartilage upon transplantation at an ectopic site. This tissue generation process requires essential stem cell characteristics including self-renewal, engraftment, proliferation, and differentiation in the microenvironment. It is necessary to assess these stemness features upon successful identification of the potential stem cell population. The conventional approaches testing MSCs isolated from bone marrow and other tissues in the Petri dish are insufficient as only a small portion are genuine SSCs (Sacchetti et al., 2007; Robey et al., 2014). The majority of MSCs lack engraftment, survival, and differentiation abilities as determined by *in vivo* transplantation analyses (Caplan and Correa, 2011; Zeitouni et al., 2012). Furthermore, the *in vitro* study cannot examine certain features of stem cell stemness, thus, missing critical criteria for the modern rigorous definition of SSCs (Bianco et al., 2013). Animal models of ectopic tissue formation have clear advantages over orthotopic transplantation because of the environments lacking interferences by cytokines and interactions with endogenous cell types, e.g., bone-forming cells (Scott et al., 2012).

For transplantation studies, the majority of studies utilize three ectopic locations: subcutaneous, intramuscular, and kidney capsule (Scott et al., 2012). The first model is subcutaneous implantation which appears to be the simplest. However, it has the most pertinent concern because of several caveats including implant migration, difficulty in identifying the implant, and notably inferior bone-forming capacity compared to alternative methods (Yang et al., 1996; Scott et al., 2012). Intramuscular implantation is the second model but there is difficulty in distinguishing the origin of cells generating the ectopic bone: donor vs. host cells. The presence of native skeletal muscle progenitors in close proximity to the implantation site may alter their fates to become bone-forming cells. This raises significant concerns about the cellular origin of the ectopic bone that could be converted from host cells by osteogenic stimulus or injury (Takaoka et al., 1988; Yu et al., 2008). This type of cell fate switching has been linked to muscle stem cell conversion from a myogenic to a fibrogenic lineage in aging mice (Brack et al., 2007). Consequently, muscle stem cells acquire the fibroblast fate, leading to muscular dystrophy (Biressi et al., 2014). Heterotopic ossification transforming cells in non-skeletal tissues into osteogenic cells is another example (Meyers et al., 2019). As a result of traumatic injury, bone formation occurs within the soft connective tissue (Shore and Kaplan, 2010). This can also be triggered by a rare congenital disease called Fibrodysplasia Ossificans Progressiva (Shore et al., 2006). Furthermore, disrupting the balance of signaling crosstalk during intramembranous ossification can alter the stem cell from osteogenic to chondrogenic fate, leading to suture fusion and craniosynostosis *via* aberrant endochondral ossification (Maruyama et al., 2010). Finally, renal capsule transplantation theoretically does not have any endogenous cell interference as the implanted cells are solely responsible for ectopic tissue generation (Scott et al., 2012). The analysis of the transplant which can be identified easily offers several advantages over the other two models.

## Renal capsule transplantation

The renal capsule environment provides the most nutrient resource for robust bone formation from significantly fewer cell numbers (Chan et al., 2015; Maruyama et al., 2016; Maruyama et al., 2021). The renal capsule lacks endogenous bone-forming cells and does not have endogenous cytokines with a negative impact on bone regeneration. The environment provides a controlled setting to test the transplanted stem cell properties. This is demonstrated by different types of bone generated by the transplantation of different stem cell sources. Cells isolated from the calvarial suture generate intramembranous-like bones while cells isolated from the tibia or femur generate endochondral-like bones (Maruyama et al., 2016; Maruyama et al., 2021). Therefore, the intrinsic characteristics of stem cells are maintained even outside of their endogenous environment. The renal capsule transplantation assay thus has the advantage to analyze stem cells and potentially examining the niche environment.

Cells to be transplanted are first counted after isolation to determine the number, followed by resuspension in the carrier/scaffold, e.g., Matrigel and Hydrogel. The cell-embedded carrier is then transferred to an insulin syringe, followed by gelation under a specific condition, e.g., temperature change and light. Next, the recipient animal is prepared by standard operations to expose the kidney. A small opening is created at the injection site using the tip of the needle. The cell-embedded carrier is directly injected underneath the outer membrane, the renal capsule region, but not inside the kidney. The transplanted kidney is then examined in 2–6 weeks after the completion of the recovery surgery. The timing of the post-operation analysis is dependent on the type of study.

## Post-transplantation analyses

**FIGURE 1:**
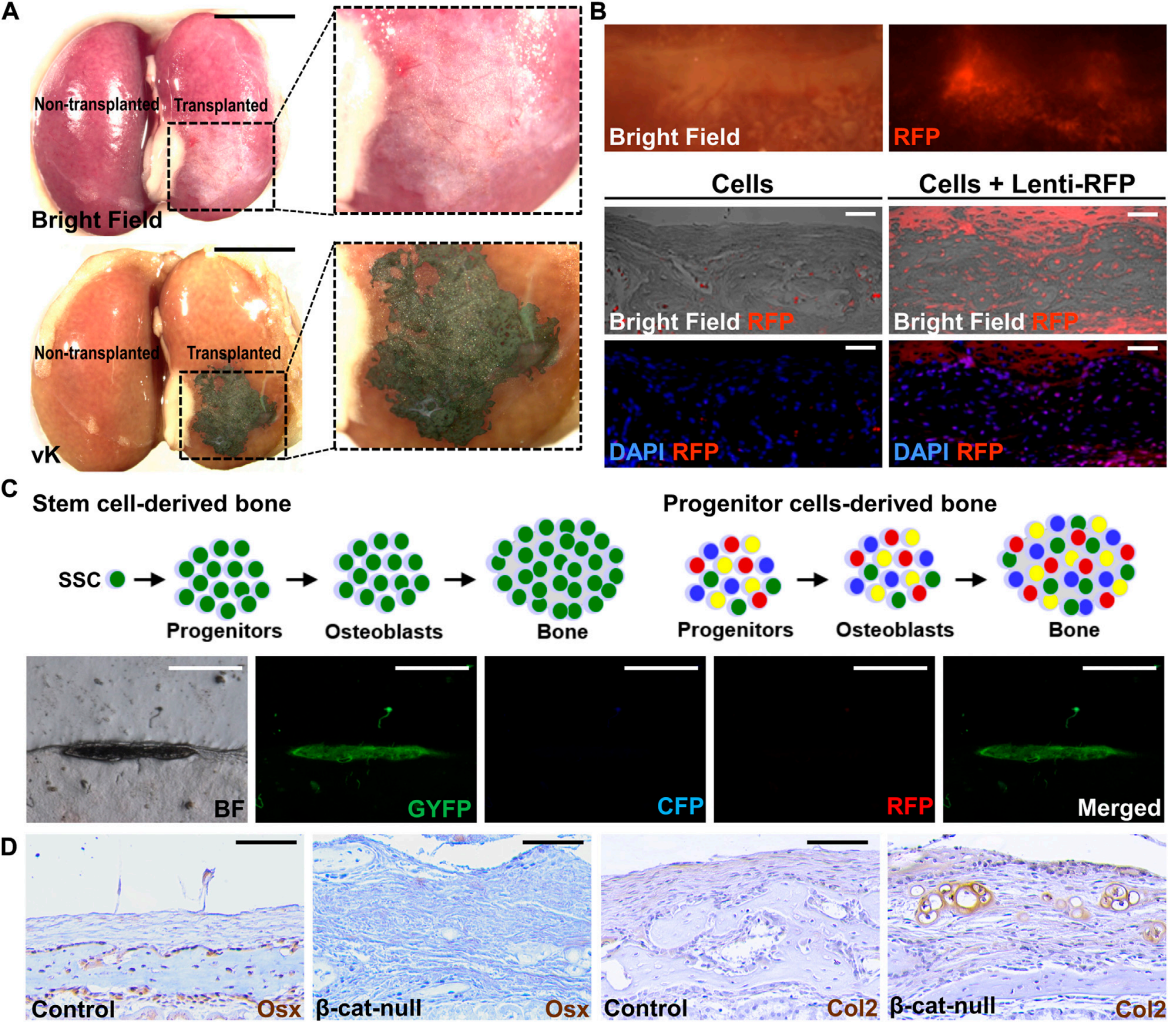
Post-transplantation analyses of tissue generation. **(A)** Wholemount analysis of the kidney transplanted (right) and non-transplanted (left) by 10^4^ cells isolated from C57/BL6 mouse sutures in bright field (top) and von Kossa (vK) staining (bottom). Enlargements of the inset indicate bone formation underneath the kidney capsule. **(B)** Wholemount analysis of the kidney transplanted by suture cells infected by lentivirus expressing the RFP reporter (Lenti-RFP). Analysis of renal sections containing the transplant of 5 × 10^4^ suture cells with or without infection of Lenti-RFP. **(C)** Diagrams illustrate the use of the confetti mouse allele to examine potential mechanisms for bone formation by the skeletal stem cell (SSC) or progenitor cells upon renal capsule transplantation. The representative images show the bone is formed by single-color cells indicating its generation by a single suture stem cell *via* the clonal expansion mechanism. **(D)** Examining lineage specification of skeletal stem cells by transplantation. Analysis of the renal capsule transplanted by 5 × 10^4^ control **(A, C)** or β-cat-null **(B, C)** suture cells by immunostaining of Osterix (Osx) and type 2 collagen (Col2). Note skeletal stem cells residing in the suture (SuSCs) are developmentally programmed to become osteoblasts positive for Osx but the loss of β-catenin alters their fate to form Col2 positive chondrocytes Scale bars, 5 mm **(A)**; 200 μm **(B)**; 400 μm **(C)**; 50 μm **(D)**.

Bone formation can be detected as early as two weeks after transplantation (Maruyama et al., 2016). Wholemount von Kossa staining offers easy identification of the mineralized tissue with even tiny bone mineralization in the renal capsule (Figure 1A). The von Kossa-positive transplants can be further examined by histology with appropriate chemical and counter-staining (Maruyama et al., 2016; Maruyama et al., 2021; Maruyama et al., 2022a). For quantitation, micro-computed tomography analysis offers a reliable method to assess the regenerated bone volume. The presence of specific cell types is then examined by immunostaining with the marker in sections. Typically, genes expressed in various stages of osteoblast differentiation, e.g., Runx2, Sp7/Osterix, Col1a1, Bglap/Osteocalcin, and Sost/Sclerostin, are used to validate osteoblastogenesis in the transplant. If the donor cells express fluorescent markers, the engraftment and ectopic tissue can be easily identified in the dissected transplants in wholemount or section (Figure 1B). The donor-specific marker further verifies tissue formation is directly attributed to the transplanted cells but to indirect effect *via* the recruitment of host cells.

Although technically more demanding, renal capsule transplantation can achieve ectopic bone generation at a single-cell level (Maruyama et al., 2016). By transplanting cells isolated from the confetti mouse model, or similar systems, e.g., Actin-CreERt Rosa26-Rainbow, to randomly label each cell with a single color (Livet et al., 2007; Ambrosi et al., 2021), the ectopic bone is formed by cells of the same color, but not by multiple colors with a mosaic pattern (Figure 1C). The findings indicate renal capsule transplantation can be used to assess the clonal expansion of SSCs *in vivo* (Maruyama et al., 2016). The renal capsule transplantation model is sensitive enough to assess stem cell numbers. The stem cell self-renewal can be determined by serial transplantation in which cells isolated from the ectopic tissue of the primary transplant are implanted again to test their regeneration ability in the secondary and subsequent transplants. These experiments highly complement the cell tracing analysis using genetic labeling system to rigorously examine adult stem cell characteristics in tissue homeostasis and regeneration.

The use of limiting dilution analysis in renal capsule transplantation has successfully assessed stem cell frequency (Maruyama et al., 2016; Maruyama et al., 2021). In this experiment, the limiting dilution analysis is performed by transplanting the number of cells with an incremental increase, e.g., 10^2^, 10^3^, 10^4^, 10^5^, and repeating various times (*n* ≥ 3), followed by the examination of ectopic bone formation. A smaller interval, e.g., 10^2^, 5 × 10^2^, 2.5 × 10^3^, 7.5 × 10^3^, theoretically can enhance the sensitivity of this assay. To determine stem cell frequency, data from the success of bone generation in the transplants are then analyzed by the ELDA software on the webpage (http://bioinf.wehi.edu.au/software/elda/). The “Estimate” indicates the stem cell frequency, thus, determining if stem cell frequency is altered by comparing control and experimental groups (*p*-value < 0.05 for statistical significance). Next, the likelihood ratio test for a single-hit model obtains the *p*-value to validate goodness of fit of the observed results.

The renal capsule transplantation model also permits the assessment of skeletal lineage specification (Chan et al., 2015; Maruyama et al., 2016; Maruyama et al., 2022a). It has been demonstrated to examine the fate alteration of the isolated SSCs (Figure 1D). The switch of SSCs from an osteogenic to a chondrogenic fate by the addition of signaling stimuli, e.g., BMP2 and VEGF inhibitor, promotes chondrogenesis (Maruyama et al., 2016; Murphy et al., 2020). Genetic inactivation of β*-catenin* in SSCs also alters their fates resulting in the generation of cartilage instead of bone (Maruyama et al., 2022a). This transplantation model thus is useful for the functional determination of factors involved in the commitment of skeletal lineages. It can also assess stem cell multipotency in an *in vivo* setting and test intrinsic defects of tissue-specific stem cells associated with fate-switching in human diseases (Maruyama et al., 2021).

## Preservation of stem cell stemness

The development of a cell culture system capable of maintaining stem cell characteristics is highly valuable for advancing our knowledge base of skeletal stem cells not only in craniofacial development and congenital deformity but especially in tissue repair and regeneration toward translational research. MSCs can be isolated from bone marrow and other tissues using conventional methods (da Silva Meirelles et al., 2006; Friedenstein et al., 1974). However, only ∼10%–20% of the isolated MSCs are genuine SSCs with self-renewing and skeletogenic abilities (Sacchetti et al., 2007; Robey et al., 2014). These MSCs also display difficulties in engraftment, survival, and differentiation of the transplanted MSCs (Caplan and Correa, 2011; Zeitouni et al., 2012). Furthermore, the cellular source of the endogenous MSC remains unknown.

**FIGURE 2:**
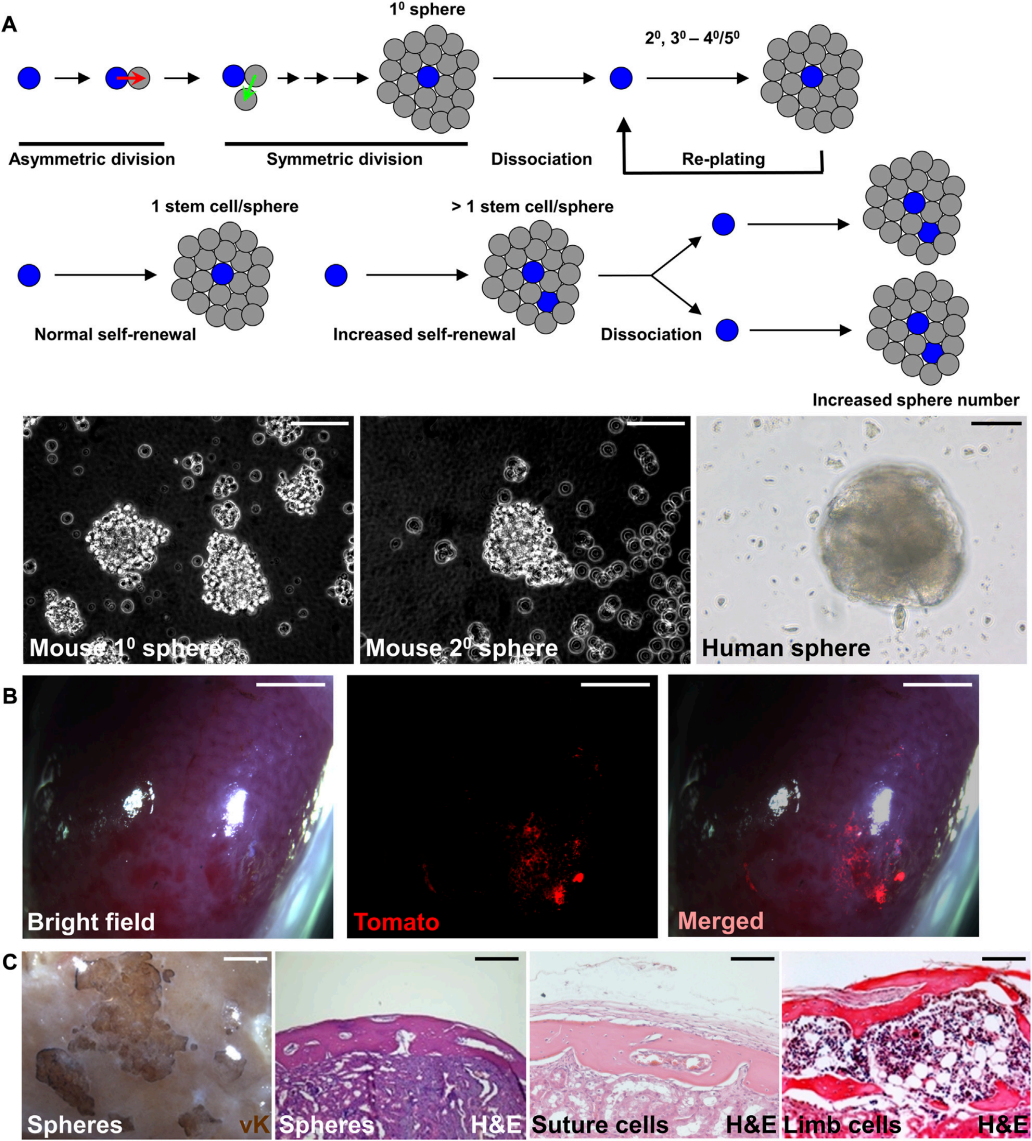
Preserving the stemness of SuSCs by sphere culture system. **(A)** Schematic representations illustrate the sphere culture of suture cells isolated from the calvarial suture mesenchyme in primary (1^0^), followed by replating and subsequent culture for secondary (2^0^), tertiary (3^0^), and up to 4^0^–5^0^ passages. The representative image of mouse and human spheres in culture. **(B)** Wholemount imaging of kidney implanted with *ex vivo* cultured spheres derived from SuSCs positive for Tomato fluorescence 4 weeks after renal capsule transplantation. **(C)** Representative images of the transplanted kidney evaluated by von Kossa (vK) and hematoxylin and eosin (H and E) staining 6 weeks after the transplantation of freshly isolated suture cells (Suture cells), or cortical cells isolated from the limb (Limb cells), or 8 weeks after the transplantation of spheres formed by the culture of suture cells (Spheres). Note bones generated by spheres show identical features to those generated by freshly isolated cells. Scale bars, 50 μm **(A)**; 2 mm **(B)**; 100 μm **(C)**.

The sphere culture method has been shown to maintain the properties of neural stem cells and mammary stem cells, recapitulating the *in vivo* characteristics (Hurley et al., 1994; Mokry et al., 1995). Similar approaches have been successfully used to establish sphere culture methods for mouse and human SuSCs (Maruyama et al., 2021; Maruyama et al., 2022b). After serial re-plating, the cultured spheres continue to form without significant decreases in number, suggesting the presence of stem cells with self-renewing ability (Figure 2A). The average sphere size remained comparable in different passages. Therefore, an increase in sphere numbers in the culture of serial passages indicates an enhanced SuSC self-renewal, thus permitting the identification of factors regulating stem cell self-renewal (Figure 2A). The current limitation for this method is up to 5 passages. At the beginning of each passage, it is essential for seeding the cells at extremely low density and on low attachment plates to avoid false positive results–a common concern for this approach containing cell clumps forming by aggregation (Maruyama et al., 2022b). The time course and cell tracing analyses further indicate a sphere is formed by the growth of a single cell (Maruyama et al., 2021).

Renal capsule transplantation of the cultured spheres can rigorously examine their osteogenic ability (Figures 2B, C). The success of bone generation at the ectopic site thus demonstrates that stem cell stemness is reserved by the sphere culture (Maruyama et al., 2021). The generated bones from the spheres also show identical features to those generated by the freshly isolated cells (Figure 2C). Histological evaluation indicates both calvarial SSCs and spheres derived from calvarial SSCs generate calvaria-like intramembranous bones while SSCs from in the tibia and femur generate bones containing large marrow cavities resembling endochondral bones (Figure 2C). In the renal capsule, the stem cells maintain their intrinsic tissue-forming/regenerating characteristics even outside of their endogenous environment (Maruyama et al., 2016; Maruyama et al., 2021). The multipotency test can examine the ability of cultured sphere cells to differentiate into osteogenic and chondrogenic cells. The renewal of stem cells can be mediated by symmetric or asymmetric division. For asymmetric mechanism, the *ex vivo* culture can be integrated into pulse-chase labeling analysis to visualize the stem cell self-renewing process as shown by the study of SuSCs (Maruyama et al., 2021). The lack of expression of proliferation markers or transient labeling of BrdU/EdU in stem cells can show the quiescent nature of SuSCs. Co-labeling of stem cell markers with potential candidate genes can determine their relevant expression patterns. In summary, the combination of renal capsule transplantation with various *in vivo* and *ex vivo* analyses provides powerful tools with clear advantages to advance skeletal stem cell research.

## Discussion

In this review, we describe recent advancements in analyses of skeletal stem cells (SSCs) using renal capsule transplantation and sphere culture systems. The combinatorial use of these systems offers additional advantages for stem cell study. Several post-transplantation assays permit the assessment of key skeletal stem cell characteristics, including stem cell frequency, clonal expansion, asymmetric division, slow-cycling/label-retaining, lineage specification, cell fate switching, engraftment, bone and cartilage formation, and stemness preservation in an *ex vivo* setting. Although suture stem cells (SuSCs) serve as an example, similar approaches, and analyses can assess SSCs from other origins. In addition to SSCs, the methods, approaches, and concepts described here can also be extended for examining other types of stem cells, thus providing powerful tools for stem cell research.
